# Wood vinegar as a natural alternative to antibiotics: Effects on cecal microbiota, antioxidant status, and nutrient digestibility in broiler chickens

**DOI:** 10.14202/vetworld.2025.1911-192

**Published:** 2025-07-17

**Authors:** Sola Samson Emmanuel, Teck Chwen Loh, Hooi Ling Foo, Henny Akit, Muhamad Faris Ab Aziz, Eric Lim Teik Chung

**Affiliations:** 1 Department of Animal Science, Faculty of Agriculture, Universiti Putra Malaysia, Serdang 43400, Selangor, Malaysia; 2 Institute of Tropical Agriculture and Food Security, Universiti Putra Malaysia, Serdang 43400, Selangor, Malaysia; 3 Department of Bioprocess Technology, Faculty of Biotechnology and Biomolecular Sciences, Universiti Putra Malaysia, Serdang 43400, Selangor, Malaysia; 4 Institute of Bioscience, Universiti Putra Malaysia, Serdang 43400, Selangor, Malaysia

**Keywords:** antibiotic alternative, antioxidant enzymes, broiler chickens, cecal microbiota, nutrient digestibility, volatile fatty acids, wood vinegar

## Abstract

**Background and Aim:**

Antibiotic resistance has spurred interest in alternative feed additives for poultry. Wood vinegar (WV), a by-product of plant pyrolysis, contains bioactive compounds with antioxidant and antimicrobial properties. This study aimed to evaluate the effects of WV supplementation through drinking water on the cecal microbial population, volatile fatty acid (VFA) concentrations, antioxidant enzyme activity, and apparent ileal nutrient digestibility in broiler chickens.

**Materials and Methods:**

A total of 432 1-day-old male Cobb 500 broiler chicks were randomly assigned to six groups (n = 72 per group; 6 replicates of 12 birds each). Treatments included a negative control (T_1_), a positive control with 0.02% oxytetracycline (T_2_), and WV-supplemented groups at dilution ratios of 1:100 (T_3_), 1:200 (T_4_), 1:500 (T_5_), and 1:1000 (T_6_) in drinking water. The experiment lasted 35 days. Plasma antioxidant enzymes (superoxide dismutase [SOD], catalase [CAT], glutathione peroxidase [GPx], total antioxidant capacity [T-AOC]), VFA profiles, ileal digestibility (crude protein [CP], ash, ether extract [EE]), and cecal microbial populations were assessed. Statistical analysis was performed using the General Linear Model and Duncan’s multiple range tests (p < 0.05).

**Results:**

WV supplementation enhanced antioxidant status, with significant increases in GPx (T_5_ and T_6_) and T-AOC (T_6_), while CAT and SOD remained unaffected. T_5_ significantly elevated acetic, butyric, and total VFA levels. WV-treated birds (T_3_–T_6_) showed reduced *Salmonella*, *Escherichia coli*, and *Enterobacteria* counts and increased bifidobacteria and total bacteria compared with controls. T_4_ showed the highest digestibility of CP, while T_5_ significantly improved ash and EE digestibility.

**Conclusion:**

WV supplementation, particularly at a 1:200 dilution (T_4_), effectively improved gut microbial balance, enhanced antioxidant enzyme activity, and promoted nutrient digestibility. These results support WV as a viable natural alternative to antibiotic growth promoters in broiler production.

## INTRODUCTION

Poultry production is increasingly challenged by environmental stressors and disease pressures that compromise the physiological function, welfare, and productivity of chickens [1, 2]. These challenges often trigger metabolic imbalances leading to excessive prod-uction of free radicals and reactive oxygen species (ROS), which damage critical biomolecules, including DNA, lipids, proteins, and carbohydrates, ultimately impairing cellular function and promoting disease onset [3, 4].

To mitigate these effects, antibiotics have histo-rically been incorporated into poultry feeds to promote growth and prevent disease [5, 6]. However, routine use of antibiotics in animal production has raised significant concerns about drug residues in meat products and the emergence of antimicrobial-resistant pathogens in humans [7, 8]. In response, regulatory bans, especially within the European Union, have restricted antibiotic use in livestock, prompting an urgent search for safer, natural alternatives that support intestinal health and productivity.

Recent research by Kareem *et al*. [9] has high-lighted the critical role of the gut microbiota in sha-ping intestinal development, immune modulation, microbial resistance, and nutrient utilization in poultry. Consequently, bioactive alternatives such as organic acids, wood vinegar (WV), plant extracts, probiotics, postbiotics, enzymes, acidifiers, and essential oils have gained traction as functional feed additives [10–13]. Among these, acidifiers have been particularly noted for their ability to lower the gastrointestinal pH, enhance nutrient absorption, and suppress the growth of patho-genic microorganisms [14].

WV, also known as pyroligneous acid, is a liquid by-product of charcoal production, composed predominantly of water (80%–90%) and a complex mixture of over 200 organic compounds (10%–20%), many of which exhibit potent antifungal, antibacterial, and antioxidant activities [15–23]. WV has been shown to inhibit pathogens such as *Salmonella* spp., *Escherichia coli*, *Bacillus subtilis*, and *Staphylococcus aureus* [24, 25]. In animal studies, dietary WV suppl-ementation has improved performance, nutrient digestibility, gut microbial balance, and meat quality, while also mitigating fecal gas emissions [26, 27]. It may also support intestinal integrity and oxidative balance, particularly in pigs [28]. The potent antib-acterial and antioxidant activities of WV are well documented [20, 29, 30]; despite these promising properties, limited data exist on the combined effects of WV on plasma antioxidant enzyme activity, cecal mic-robial community, and digestibility in broiler chickens.

While the antimicrobial and antioxidant prop-erties of WV have been widely acknowledged acr-oss various animal models, including pigs and fish, its application in poultry nutrition remains relatively underexplored. Existing studies have largely focused on WV’s effect on growth performance and meat quality, with less emphasis on its role in modulating intestinal health parameters such as gut microbiota composition, volatile fatty acid (VFA) production, and nutrient absorption in broiler chickens. In addition, the influence of WV on systemic oxidative stress markers, especially plasma antioxidant enzyme activity, has not been comprehensively studied in broilers. Given that gut health and oxidative balance are critical for optimal poultry productivity and disease resistance, the absence of integrated data addressing these biological endpoints presents a significant knowledge gap. Moreover, the dose–response relationship of WV supplementation in drinking water and its comparative efficacy with conventional antibiotic treatments in broiler chickens remains insufficiently characterized in the literature.

This study was designed to comprehensively evaluate the effects of graded levels of WV supple-mentation in drinking water on key physiological and intestinal health parameters in broiler chickens. Specifically, the research aimed to (i) assess the impact of WV on the composition of cecal microbiota, including both beneficial and pathogenic bacterial populations; (ii) quantify changes in cecal VFA profiles as indicators of microbial fermentation activity; (iii) measure plasma antioxidant enzyme activity – namely glutathione pero-xidase (GPx), superoxide dismutase (SOD), catalase (CAT), and total antioxidant capacity (T-AOC) – to dete-rmine systemic oxidative balance; and (iv) evaluate the apparent ileal digestibility (AID) of crude protein (CP), ether extract (EE), and ash to determine nutrient utilization efficiency. By examining these parameters in tandem, the study sought to determine whether WV could serve as a natural and effective alternative to antibiotics, thereby contributing to the development of sustainable poultry production strategies.

## MATERIALS AND METHODS

### Ethical approval

The study was approved by the Institutional Animal Care and Use Committee (IACUC) under reference num-ber UPM/IACUC/AUP-R05/2022.

### Study period and location

The study was conducted from September to November 2022 at the Department of Animal Science, Faculty of Agriculture, Universiti Putra Malaysia, Ser-dang, 43400, Selangor, Malaysia

### Experimental design and animal management

A total of 432 one-day-old male broiler chicks (Cobb 500) were procured from a commercial supplier. Upon arrival, the chicks were individually tagged, weighed, and randomly allocated to six treatment groups using a completely randomized design. Each treatment group consisted of six replicates with 12 chicks per replicate.

The birds were reared in a closed-house system with a controlled initial temperature of 33°C ± 1°C, which was gradually reduced to approximately 25°C ± 1°C by day 15. Relative humidity was maintained between 60% and 70% throughout the experiment. Feed and water were provided *ad libitum* during the entire 35-day trial.

All birds were fed a common basal diet, and the treatment differences were applied only to the drinking water. The feeding schedule was divided into three phases: Starter (days 0–14), grower (days 15–28), and finisher (days 29–35). The experimental diets are det-ailed in Table 1.

**Table 1:** Nutrient composition of starter, grower, and finisher diets.

Ingredients	Starter	Grower	Finisher
Corn	54.00	58.36	57.20
Soybean meal	36.50	29.80	27.10
Wheat pollard	3.77	7.10	6.27
Palm oil	0.70	0.70	2.83
Lysine	0.27	0.34	0.31
Dicalcium phosphate 18%	3.30	1.90	1.70
Salt	0.35	0.35	0.35
Toxin binder	0.10	0.10	0.10
Mineral mix	0.15	0.15	0.15
Choline chloride	0.10	0.10	0.10
Vitamin mix	0.15	0.15	0.15
Methionine	0.36	0.35	0.33
Antioxidant	0.10	0.10	0.10
Threonine	0.15	0.16	0.12
Calcium carbonate	-	0.30	0.28
L-arginine	-	0.04	0.05
Palm kernel cake	-	-	2.86
Total	100.00	100.00	100.00
Calculated analysis			
Metabolizable energy (Kcal/kg)	2903.11	2,950.72	3050.52
Protein (%)	21.08	19.05	18.03
Fat (%)	2.99	3.11	5.37
Fiber (%)	4.20	4.09	4.23
Calcium (%)	1.15	0.81	0.74
Total phosphorus (%)	1.05	0.79	0.80
Available phosphorus (%)	0.60	0.41	0.37

Vitamin premix supplied/diet Kg. Vitamins=(22 g=B2, 90 g=E, 7 g=B1, 35 Million International Unit (MIU) A, 6 g=K3, 0.070 g=B12, 9 MIU=D3, 12 g=B6, 120 g=Nicotinic acid, 300 mg=Biotin, 3 g=B9, 35 g=Pantothenic acid, 25000 FTU=Phytase). Per kg of diet, mineral mix supplied. Zn=80, Fe=80 g, Na=1.5 g, I=1 g, Cu=15 g, Mn=100 g, K=4 g, Se=0.2 g, Co=0.25 g

The six experimental groups were defined as follows:

- T_1_: Basal diet + plain water (negative control)
- T_2_: Basal diet + 0.02% (w/v) oxytetracycline in drinking water (positive control)
- T_3_: Basal diet + 1:100 (v/v) WV in drinking water
- T_4_: Basal diet + 1:200 (v/v) WV
- T_5_: Basal diet + 1:500 (v/v) WV
- T_6_: Basal diet + 1:1000 (v/v) WV

The WV used in this study was derived from bamboo and administered through drinking water.

### Sample collection and handling

At the end of the 35-day feeding trial, six broiler chickens from each treatment group were randomly selected and slaughtered in accordance with halal guidelines outlined in MIS500:2009 [31]. Blood sam-ples were collected through venipuncture into ethyle-nediaminetetraacetic acid-coated vacutainer tubes (BD Vacutainer, New Jersey, USA), maintained on ice, and centrifuged at 3500 × *g* for 15 min at 4°C. The plasma was then separated and stored at −80°C for sub-sequent antioxidant enzyme analysis.

Ileal digesta was collected to determine nutrient digestibility, while cecal chyme was harvested for VFA analysis and microbial quantification [32].

### Plasma antioxidant enzyme activity

Plasma activities of SOD, CAT, and GPX were assessed using EnzyChrom™ assay kits (Bioassay Systems, Hayward, CA, USA) and T-AOC was measured using the QuantiChrom™ assay kit (Elabscience, E-BC-K219, Houston, TX, USA), following the manufacturer’s instru-ctions. Detailed protocols are as previously reported by Azizi *et al*. [33].

T-AOC was measured based on the reduction of Cu^2+^ to Cu^2+^ which then forms a colored complex with a dye reagent; absorbance was directly proportional to antioxidant capacity. GPx activity was quantified based on the oxidation of nicotinamide adenine dinucleotide phosphate at 340 nm. CAT activity was measured thro-ugh hydrogen peroxide degradation using a redox dye, and SOD activity was evaluated by monitoring the red-uction of superoxide (O_2_^-^) using the WST-1 dye. All abs-orbance readings were taken using a Multiskan™ Go spectrophotometer (Thermo Scientific, MA, USA).

### VFA analysis

Cecal VFA concentrations were determined following a modified method described in Kareem *et al*. [34]. Approximately 1 g of cecal content was mixed with 1 mL of 24% metaphosphoric acid in 1.5 molar sulfuric acid and left overnight at 25°C. Samples were centrifuged at 10,000 × *g* for 20 min at 4°C, and 0.5 mL of the supernatant was transferred into a 1.5 mL glass vial containing 0.5 mL of 20 mM 4-methyl-valeric acid (internal standard).

VFA separation was conducted using a Quadrex 007 gas chromatography (GC) system (Quadrex Corp., New Haven, CT 06525, USA), with a fused silica capillary column (15 m × 0.32 mm inner diameter, 0.25 µm film thickness) and a flame ionization detector. Liquid nitrogen was used as the carrier gas at a flow rate of 60 mL/min. The column temperature was set at 200°C, and both injector and detector temperatures were maintained at 230°C. VFA peaks were identified using commercial standards, and the GC was calibrated internally for accuracy

### Cecal microbial population quantification

Cecal contents were aseptically collected, snap-frozen in liquid nitrogen, and stored at −80°C until analysis. The cecal microbial populations were quantified following the procedure outlined by Humam *et al*. [35]. DNA was extracted using the QIAamp DNA Stool Mini Kit (Qiagen, Hilden, Germany) according to the manufacturer’s protocol. Approximately 200 mg of cecal content was treated with InhibitEX buffer, heated at 95°C, vortexed, and centrifuged. The supernatant was subjected to enzymatic digestion with Proteinase K and buffer AL, followed by ethanol precipitation and purification using QIAamp spin columns. The final DNA was eluted in 50 μL ATE buffer and stored at −20°C. DNA concentration and purity were determined by a NanoDrop 2000 spectrophotometer (Thermo Scientific Wilmington, DE, USA) before quantitative polymerase chain reaction (qPCR).

qPCR was conducted using CAPITAL™ qPCR Green Master Mix (Biotechrabbit GmbH, Neuendorfstraße 24a, 16761 Hennigsdorf, Germany) with 1 μL each of forward and reverse primers, 1 μL DNA template, and RNase-free water to a final volume of 20 μL. Amplification was performed using a BioRad CFX96 real-time PCR (Bio-Rad Hercules, California, USA) system with the following thermal conditions: Initial denaturation at 95°C for 30 s; 40 cycles of denaturation at 95°C for 5 s; annealing at primer-specific temperatures (58°C for *Lactobacillus*, 55°C for total bacteria, 60°C for *Bifidobacterium*, and 50°C for *E. coli*, *Enterobacteriaceae*, *Enterococcus*, and *Salmonella*); and extension at 72°C for 20 s. Primer sequences and target bacteria are listed in Table 2 [36–39].

**Table 2:** Primer sequences used for quantitative PCR of cecal microbial populations in broiler chickens.

Target microbe	Primer sequence 5’–3’	Product size (bp)	Reference
Total bacteria	CGGCAACGAGCGCAACCC–F CCATTGTAGCACGTGTGTAGCC–R	145	[36]
*Enterococcus*	CCCTTATTGTTAGTTGCCATCATT–F ACTCGTTGTACTTCCCATTGT–R	144	[36]
*Bifidobacteria*	GGGTGGTAATGCCGGATG–F TAAGCCATGGACTTTCACACC–R	278	[37]
*Lactobacillus*	CATCCAGTGCAAACCTAAGAG–F GATCCGCTTGCCTTCGCA–R	341	[38]
*Salmonella*	TCGTCATTCCATTACCTACC–F AAACGTTGAAAAACTGAGGA–R	119	[39]
*E. coli*	GTGTGATATCTACCCGCTTCGC–F AGAACGCTTTGTGGTTAATCAGGA–R	82	[38]
*Enterobacteria*	CATTGACGTTACCCGCAGAAGAAGC–F CTCTACGAGACTCAAGCTTGC–R	195	[38]

R=Reverse; F=Forward; bp=Base pair

### Apparent ileal digestibility of nutrients

The AID of CP, dry matter (DM), EE, and ash was calculated using titanium dioxide (TiO_2_) as an indigestible marker, following the method of Short *et al*. [40]. Appr-oximately 0.1 g of ileal digesta was ashed at 580°C for 13 h, cooled, and digested in 10 mL of 7.4 M H_2_SO_4_. The mixture was boiled for 60 min, diluted with 25 mL of distilled water, filtered using Whatman No. 541 filter paper, and transferred into a 100 mL volumetric flask.

To each flask, 20 mL of 30% hydrogen peroxide (H_2_O_2_) was added and the volume was adjusted to 100 mL with distilled water. A 0.3 mg/mL working standard of TiO_2_ was prepared by dissolving 150 mg TiO_2_ in 100 mL of H_2_SO_4_, boiling, and diluting to 500 mL. A standard curve was generated using solutions ranging from 0 to 10 mL. Absorbance was measured at 410 nm using a spectrophotometer. Digestibility coefficients were computed using standard formulas as reported by Goh *et al*. [41].


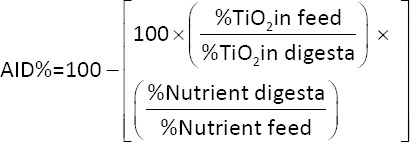


### Statistical analysis

Statistical analyses were performed using the General Linear Model (GLM) of SAS software [42] version 9.4 (SAS Institute Inc., Cary, NC, USA) using one-way analysis of variance. Treatment means were compared using Duncan’s multiple range test, and significance was accepted at p < 0.05 [39]. Linear and quadratic responses to increasing WV concentrations were evaluated using orthogonal polynomial contrasts. The statistical model was Y_ijk_ = μ+T_ij_+E_ijk_ where Y_ijk_ is the dependent variable, μ is the general mean, T_ij_ is the effect of WV, and E_ijk_ is the experimental error.

## RESULTS

### Antioxidant enzyme activity

The activities of CAT and SOD were not significantly affected (p > 0.05) across the treatment groups. In contrast, T-AOC was significantly increased (p < 0.05) in all WV-treated groups compared with the T_1_ and T_2_ groups, with T_6_ exhibiting the highest T-AOC activity. GPx activity was also significantly elevated (p < 0.05) in the T_5_ and T_6_ groups relative to the other treatment groups, whereas T_1_ recorded the lowest GPx activity among all groups (Table 3).

**Table 3:** Antioxidant enzyme activity in broiler chickens supplemented with WV and drinking water.

Parameters	Treatments						^1^SEM	p-value	^2^Contrast p-value	
	
	T_1_	T_2_	T_3_	T_4_	T_5_	T_6_			Linear	Quadratic
T-AOC (µM)	373.41^c^	404.36^b^	425.79^b^	428.17^ab^	428.17^ab^	435.31^a^	6.03	0.0037	0.053	0.0040
GPX (µmol/L)	231.07^c^	271.05^c^	308.10^bc^	357.98^b^	461.78^a^	421.43^a^	22.54	0.0020	0.4102	0.0005
SOD (U/mL)	2.22	2.20	2.71	2.44	2.51	2.24	0.13	0.8742	0.2425	0.8831
CAT (U/mL)	23.75	27.37	33.36	29.53	37.18	28.16	1.67	0.2435	0.1277	0.2519

Mean values in a row with dissimilar alphabets (a,b,c) as superscripts vary substantially (p < 0.05). T_1_=Negative control, T_2_=Positive control, T_3_=1:100 (v/v) WV, T_4_=1:200 (v/v) WV, T_5_=1:500 (v/v) WV, T_6_=1:100 (v/v) WV, CAT=Catalase, T-AOC=Total antioxidant capacity, SOD=superoxide dismutase, GPx=Glutathione peroxidase,

1 SEM=Standard error of mean,

2 Contrast p*-*values=Linear and quadratic response determined with orthogonal polynomial contrast, WV=Wood vinegar

### VFA profile

The effects of WV supplementation on cecal VFA concentrations are summarized in Table 4. Acetic acid production was significantly higher (p < 0.05) in the T_3_ and T_5_ groups compared to the control groups (T_1_ and T_2_). However, no significant differences (p > 0.05) were observed in acetic acid concentrations among the T_4_, T_6_, and control groups.

**Table 4:** Concentrations of cecal volatile fatty acids in broiler chickens supplemented with WV windring water.

Parameters	Treatments						^1^SEM	p-value	^2^Contrast p-value	
	
	T_1_	T_2_	T_3_	T_4_	T_5_	T_6_			Linear	Quadratic
Acetic acid	33.55^b^	33.60^b^	44.29^a^	34.74^b^	48.67^a^	36.79^b^	1.33	0.0002	0.0082	0.1118
Propionic acid	1.11^c^	2.34^b^	2.38^b^	2.35^b^	2.85^b^	3.97^a^	0.17	<0.0001	0.1666	<0.0001
Butyric acid	3.50^e^	4.76^d^	6.24^b^	5.61^c^	7.70^a^	5.25^cd^	0.24	<0.0001	<0.0001	<0.0001
Others	0.70	1.34	1.23	0.86	1.51	1.44	0.09	0.0626	0.5207	0.0966
Total VFA	38.87^d^	42.04^cd^	54.13^b^	43.55^cd^	60.72^a^	47.45^c^	1.63	<0.0001	0.0025	0.0092

Mean values in a row with dissimilar alphabets (a,b,c,d) as superscripts vary substantially (p < 0.05). T_1_=Negative control, T_2_=Positive control, T_3_=1:100 (v/v) WV, T_4_=1:200 (v/v) WV, T_5_=1:500 (v/v) WV, T_6_=1:1000 (v/v) WV,

1 SEM=Standard error of mean,

2 Contrast p*-*values=Linear and quadratic response determined with orthogonal polynomial contrast, WV=Wood vinegar

Propionic acid levels were significantly elevated in the T_6_ group, whereas T_1_ had the lowest concentrations. The propionic acid values in T_3_, T_4_, and T_5_ were similar to T_2_ but were significantly higher than those in T_1_. Butyric acid production was significantly enhanced (p < 0.05) in the T_5_ group. Similarly, the total VFA concentration showed a significant linear and quadratic trend in T_5_. Other VFAs – including isobutyric, isovaleric, and valeric acids – did not differ significantly (p > 0.05) across treatments.

### Cecal microbial population

**Figure 1:**
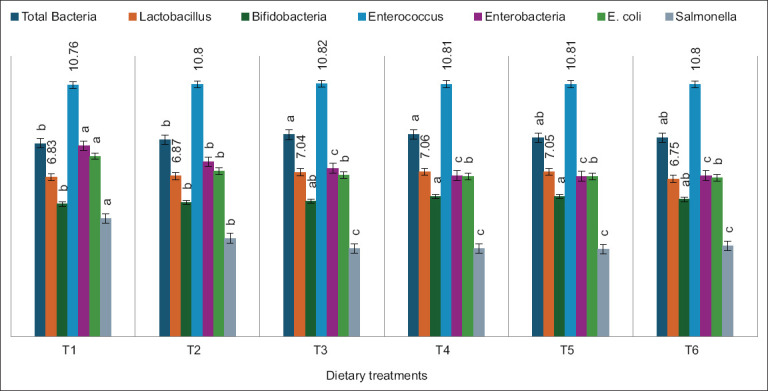
Effect of WV on cecal microbial population of broiler chickens. Different letters on standard error bars indicate significant difference (p < 0.05). T_1_=Negative control, T_2_=Positive control, T_3_=1:100 (v/v) WV, T_4_=1:200 (v/v) WV, T_5_=1:500 (v/v) WV, T_6_=1:1000 (v/v) WV, WV=Wood vinegar.

The effects of WV supplementation on the cecal microbial population of broiler chickens are illustrated in Figure 1. The total bacterial population was significantly higher (p < 0.05) in the T_3_ and T_4_ groups compared to the control group (T_1_). However, the populations of *Enterococcus* and *Lactobacillus* were not significantly influenced by treatment.

WV supplementation significantly increased (p < 0.05) the population of *Bifidobacterium* spp., with T_4_ and T_5_ groups showing the highest counts relative to T_1_ and T_2_ groups. In contrast, *E. coli* levels exhibited a significant linear and quadratic increase in T_1_, while all WV-treated groups had reduced *E. coli* populations. Similarly, *Salmonella* counts were significantly higher (p < 0.05) in T_1_ and declined across WV-treated groups (T_3_, T_4_, T_5_, and T_6_). The *Enterobacteria* population was also significantly higher in the T_1_ group compared to T_3_, T_4_, T_5_, and T_6_.

### Nutrient digestibility

WV supplementation had no significant effect (p > 0.05) on DM digestibility. However, CP, ash, and EE digestibility were significantly influenced (p < 0.05) by the treatments. CP digestibility was highest in the T_4_ group, whereas T_1_ had the lowest. Ash digestibility increased significantly in a linear and quadratic manner in the T_5_ group. EE digestibility was significantly higher in all WV-treated groups compared to T_1_ (Table 5).

**Table 5:** Nutrient digestibility of broiler chickens supplemented with WV and drinking water.

Parameters	Treatments						^1^SEM	p-value	^2^Contrast p-value	
	
	T_1_	T_2_	T_3_	T_4_	T_5_	T_6_			Linear	Quadratic
Dry matter	67.08	67.04	67.00	67.17	67.17	67.00	0.15	0.1897	0.5624	0.6434
Ash	36.64^d^	36.41^d^	40.09^c^	45.27^b^	46.96^a^	42.80^b^	0.77	<0.0001	<0.0001	0.0037
Ether extract	61.90^c^	64.59^c^	70.24^b^	73.87^a^	72.77^a^	72.46^a^	2.07	0.0199	0.0017	0.0814
Crude protein	68.05^e^	71.26^d^	75.59^c^	80.84^a^	76.46^b^	74.19^c^	0.60	<0.0001	0.5723	0.7925

Mean values in a row with dissimilar alphabets (a,b,c, and d) as superscripts vary significantly (p < 0.05). T_1_=Negative control, T_2_=Positive control, T_3_=1:100 (v/v) WV, T_4_=1:200 (v/v) WV, T_5_=1:500 (v/v) WV, T_6_=1:1000 (v/v) WV,

1 SEM=Standard error of mean,

2 Contrast p*-*values=Linear and quadratic response determined with orthogonal polynomial contrast, WV=Wood vinegar

## DISCUSSION

### Antioxidant enzyme activity

Oxidative stress occurs when there is an imba-lance between ROS and the body’s antioxidant defense mechanisms. The accumulation of ROS can damage essential biomolecules, including proteins, DNA, and lipids, leading to cellular injury [43]. Enhancing antioxidant capacity is therefore critical to maintaining redox balance and physiological function in poultry. As reported by Geret *et al*. [44], GPx, CAT, and SOD are key antioxidant enzymes that serve as the body’s primary defense against ROS. According to Azizi *et al*. [32], both GPx and SOD are essential in mitigating free radical-induced damage, while CAT specifically degrades H_2_O_2_ into oxygen and water, protecting cells from oxidative harm [45].

T-AOC is a comprehensive indicator of the cumu-lative activity of all antioxidants within the body [43]. Thus, T-AOC, along with SOD and CAT, is a vital parameter for assessing systemic oxidative resistance [46].

In the present study, WV supplementation resulted in a marked improvement in GPx and T-AOC activities, particularly at higher concentrations, whereas CAT and SOD activities remained unchanged. This aligns with the findings of Wang *et al*. [47], who reported similar selective modulation of antioxidant enzymes. The observed effects may be attributed to phenolic compounds and organic acids in WV, which enhance the body’s oxidative stability [15, 20, 48]. WV acts as a natural antioxidant and microbial inhibitor, helping to reduce oxidative stress and enhance animal health [20]. By suppressing ROS generation, WV potentially miti-gates cellular damage and supports overall well-being in broilers [49]. The unchanged CAT and SOD activities may reflect the generally optimal health and management conditions during the trial, which may have sustained baseline antioxidant enzyme levels [48].

### VFAs

Short-chain fatty acids (SCFAs), such as butyric, propionic, and acetic acids, are produced during the microbial fermentation of carbohydrates in the avian gut and serve as key indicators of fermentation efficiency and microbial activity [50]. The present study demonstrated that WV supplementation increased acetic, propionic, butyric, and total VFA concentrations compared with the negative control, especially in the T_5_ group.

WV likely promotes VFA production by enha-ncing the availability of nutrients for microbial ferme-ntation and by increasing the abundance of beneficial microbes. Notably, acetic acid, identified as the primary organic compound in WV [51], was also found at the highest concentrations among all VFAs in this study, confirming earlier observations by Han *et al*. [52]. These findings are consistent with Kareem *et al*. [34], who reported elevated VFA levels in broilers following suppl-ementation with postbiotics, and with Goiri *et al*. [53], who observed similar effects with biochar in the diet.

The cecum is the primary site of microbial fermentation in poultry, where indigestible carboh-ydrates are metabolized into SCFAs and gases [54]. VFAs help lower intestinal pH, which discourages the growth of pathogenic organisms [55]. The observed increase in total VFAs likely reflects improved microbial fermentation and an enriched population of beneficial bacteria such as *Bifidobacterium* and *Lactobacillus*, potentially explaining the increased acetic acid levels. To date, limited studies have examined the effect of WV on VFAs in broilers, highlighting the novelty of these findings.

### Cecal microbial population

WV supplementation promoted a general increase in total bacterial populations while concurrently supp-ressing pathogenic bacteria in the cecal digesta of broiler chickens. The cecum is the most microbially dense section of the poultry gut and plays a crucial role in nutrient metabolism, immune regulation, and maintaining microbial balance [56, 57]. Its favorable environment supports the proliferation of commensal bacteria, which enhance animal health and produ-ctivity [58, 59].

The increase in beneficial microbial populations observed in WV-treated groups may be attributed to a reduction in intestinal pH through acetic acid elevation, thereby creating an unfavorable environment for pathogens [59]. Organic acids – including those present in WV – can penetrate bacterial cell membranes and disrupt their function, limiting colonization and infection risk [51, 60]. WV, being a natural source of organic acids, has been shown to modulate gut microbiota composition by promoting beneficial bacteria and reducing pathogen burden [61].

These findings align with previous studies by Kareem [34] and Muhammad *et al*. [62], which reported reduced bacterial loads and enhanced populations of *Lactobacillus* and *Bifidobacterium* following postbiotics or organic acid supplementation in broilers. Although differences in *Lactobacillus* abundance were not stati-stically significant across treatments, a numerical increase was observed in the WV groups, suggesting a positive modulatory effect. According to Dibner *et al*. [63], WV inclusion suppresses pathogens while improving beneficial bacterial populations such as *Enterococcus* and *Bifidobacterium*. Similar findings have been reported in pigs and ducks [64–66], while Saleem *et al*. [67] found reduced bacterial counts following organic acid supplementation in poultry.

Contrastingly, Hanchai *et al*. [59] found no effect of WV on chicken gut flora, indicating potential variability based on experimental conditions. Nonetheless, the present study supports findings from Yan *et al*. [64] and Watarai and Tana [68] that WV exerts both anti-microbial and probiotic-like actions, enhancing gut health by reducing harmful bacteria and supporting the develo-pment of beneficial microbes.

### Nutrient digestibility

WV supplementation improved the AID of CP, EE, and ash. This enhancement may be attributed to WV’s ability to reduce gastrointestinal pH, thereby facilitating optimal enzyme activity and nutrient breakdown. Organic acids are known to support nutrient digestibility by modulating intestinal pH and suppressing microbial competition for nutrients [26].

Studies by Choi *et al*. [26] and Yan *et al*. [64] have shown that dietary WV inclusion at 0.1%–0.3% improves nutrient digestibility in pigs, and Sureshkumar *et al*. [69] demonstrated similar effects in grower-finisher pigs. The present findings are also in agreement with Khooshechin *et al*. [70], who reported improved ileal digestibility in broilers fed diets supplemented with organic acids. These effects are likely due to the antimicrobial and acidifying properties of WV, which stabilize gut conditions and enhance nutrient utili-zation [69].

Organic acids have also been reported to improve nutrient absorption and gut health in broilers by lowering gut pH and inhibiting the growth of harmful microbes [71, 72]. WV shares these functional prop-erties, and its supplementation has been linked to enhanced digestion and absorption of key nutrients [73]. According to Ju *et al*. [74], dietary WV may reduce pathogenic bacteria by modulating gut pH, which in turn supports better nutrient digestibility. Therefore, the improved nutrient utilization observed in this study may stem from WV’s regulatory effects on gut microbial composition and gastrointestinal pH.

## CONCLUSION

This study demonstrated that dietary supplem-entation of WV through drinking water had a positive influence on antioxidant status, cecal microbial popu-lations, VFA production, and nutrient digestibility in broiler chickens. Specifically, WV at 1:200 (v/v) and 1:500 (v/v) dilutions significantly enhanced GPx and T-AOC without adversely affecting CAT or SOD. In addition, WV supplementation led to increased concentrations of acetic, propionic, and butyric acids in the cecum, with the T_5_ group showing the most pronounced effect. WV-treated groups also exhibited a significant increase in beneficial bacteria (*Bifidobacterium* spp. and total bacteria) and a marked reduction in pathogenic bacteria (*Salmonella*, *E. coli*, and *Enterobacteriaceae*). Apparent ileal digestibility of CP, ash, and EE was notably improved, particularly in the T_4_ and T_5_ groups.

From a practical standpoint, these findings suggest that WV can serve as an effective natural alternative to in-feed antibiotics, promoting gut health, microbial balance, and nutrient utilization without compromising oxidative stability. Its ease of administration through drinking water and dose-dependent efficacy make WV a feasible option for sustainable poultry production systems, especially in antibiotic-restricted or antibiotic-free environments.

A major strength of this study lies in its integrated assessment of physiological, microbial, and nutritional outcomes using a well-structured randomized design and multiple WV inclusion levels. However, one limitation is that the study did not evaluate morphological changes in gut histology or the expression of immune-related genes, which could further elucidate the mechanistic effects of WV.

Future research should investigate the long-term impacts of WV on broiler health, meat quality, gut morphology, and immune gene expression under commercial field conditions. Studies exploring the synergistic effects of WV with other phytogenic or postbiotic compounds could also enhance its application in precision poultry nutrition.

In conclusion, WV supplementation – particularly at a concentration of 1:200 (v/v) – holds strong potential as a multifunctional feed additive that supports oxidative balance, improves gut microbiota, and enhances nut-rient digestibility, thereby contributing to more resilient and sustainable poultry farming practices.

## DATA AVAILABILITY

All the generated data are included in the manuscript.

## AUTHOR’S CONTRIBUTIONS

SSE, TCL, and HLF: Conceptualized the study. TCL, HLF, HA, MFA, and ELTC: Supervised the study and reviewed and edited the manuscript. SSE, TCL, and MFA: Statistical analysis. SSE: Drafted the manuscript. All authors have read and approved the final manuscript.
